# Incidence and Risk Factors of Postoperative Complications in Patients Undergoing Robot-assisted Laparoscopic Radical Prostatectomy: A Retrospective Study

**DOI:** 10.4274/TJAR.2025.251973

**Published:** 2025-12-22

**Authors:** Oya Kılcı, Feryal Korkmaz Akçay, Özlem Balkız Soyal, Murat Akçay, Betül Güven Aytaç

**Affiliations:** 1 Ankara Bilkent City Hospital Clinic of Anaesthesiology and Reanimation, Ankara, Türkiye

**Keywords:** Anaesthetic complications, perioperative care, postoperative care unit, risk factors, robotic prostate surgery

## Abstract

**Objective:**

Robot-assisted laparoscopic radical prostatectomy (RALP) is increasingly used in the treatment of prostate cancer due to its minimally invasive nature, reduced perioperative bleeding, and shorter hospital stays. However, the steep Trendelenburg position and CO₂ pneumoperitoneum required for the procedure present unique anaesthetic challenges, particularly in elderly patients with comorbidities. This study aimed to determine the incidence of anaesthetic complications during RALP and identify independent risk factors associated with these events.

**Methods:**

A retrospective observational study was conducted at Ankara Bilkent City Hospital between 2019 and 2024. A total of 1,020 patients who underwent RALP were evaluated. Collected data included demographic characteristics, the American Society of Anesthesiologists (ASA) physical status classification, comorbidities, and intra- and postoperative outcomes. Anaesthetic complications were analyzed, and multivariate logistic regression was performed to identify independent predictors.

**Results:**

The mean patient age was 65.0±6.3 years, with 65.3% classified as ASA II and 61.6% having at least one comorbidity. Anaesthetic complications occurred in 4.4% of patients. Those with complications were significantly older (67.9±6.2 vs. 64.9±6.3 years, *P*=0.004), had longer hospital stays (8.98±4.45 vs. 6.83±3.18 days, *P* < 0.001), and were more frequently admitted to the post-anaesthesia care unit (PACU) (73.3% vs. 46.8%, *P* < 0.001). Multivariate analysis identified age, hospital stay duration, and PACU admission as independent risk factors.

**Conclusion:**

RALP can be safely performed in experienced centers with individualized anaesthetic management. However, older age, longer hospitalization, and PACU admission significantly increase the risk of anaesthetic complications. These findings emphasize the need for preoperative risk stratification and tailored perioperative care to improve safety outcomes. Prospective, multicenter studies are needed to confirm these results and guide future anaesthetic strategies in robotic urologic surgery.

**Main Points**

• The study analyzed 1,020 patients undergoing robot-assisted laparoscopic radical prostatectomy (RALP) with a mean age of 65 years, most classified as the American Society of Anesthesiologists II, indicating significant comorbidities.

• Anaesthetic complications were observed in 4.4% of cases, emphasizing the need for vigilance in managing these patients.

• Multivariate analysis identified age, prolonged hospital stay, and post-anaesthesia care unit (PACU) admission as independent risk factors for anaesthetic complications.

• Every additional year of age increased complication risk by 1.083 times, while each extra day of hospitalization raised it by 1.128 times. PACU admission led to over a three-fold increase in risk.

• These findings highlight the need for thorough preoperative evaluation and tailored anaesthetic management to improve safety in RALP procedures.

## Introduction

Robot-assisted laparoscopic radical prostatectomy (RALP) has become a widely adopted technique for prostate cancer due to its minimally invasive nature and perioperative benefits, including reduced blood loss, shorter operative times, and decreased length of hospital stay compared to open surgery. However, RALP poses distinct anaesthetic challenges primarily due to the combined effects of steep Trendelenburg positioning and CO₂ pneumoperitoneum, which induce significant physiological changes. The steep Trendelenburg position shifts abdominal contents toward the diaphragm, resulting in reduced lung volumes, impaired pulmonary compliance, and increased airway pressures. These alterations compromise respiratory mechanics and may lead to atelectasis, ventilation-perfusion mismatch, and increased work of breathing. Simultaneously, CO₂ pneumoperitoneum elevates intra-abdominal pressure, which decreases functional residual capacity and promotes hypercapnia and respiratory acidosis.^1^ These effects are particularly pronounced in elderly patients and those with underlying pulmonary disease, such as chronic obstructive pulmonary disease (COPD), thereby increasing the risk of perioperative pulmonary complications. Furthermore, elevated intrathoracic and intracranial pressures associated with positioning and pneumoperitoneum may result in hemodynamic instability, airway edema, and neurological sequelae.^2^ The cumulative impact of these factors may help explain the observed association between increased age, post-anaesthesia care unit (PACU) admission, and anaesthetic complications in our patient cohort.

Given the physiological challenges associated with RALP, anaesthesiologists should carefully manage ventilation strategies to reduce the risk of hypercapnia, hypoxemia, and other respiratory complications. Pressure-controlled ventilation has been reported to lower peak airway pressures and improve dynamic compliance compared with volume-controlled ventilation, although both methods are commonly used. Additionally, positive end-expiratory pressure (PEEP) can help maintain adequate oxygenation during prolonged pneumoperitoneum.^3^

Patients undergoing RALP are often older and can have several comorbidities, such as hypertension, diabetes mellitus, coronary artery disease (CAD), and renal dysfunction. These factors can complicate anaesthetic management and increase the risk of perioperative complications. The American Society of Anesthesiologists (ASA) physical status classification system is commonly used to evaluate preoperative risk, and higher ASA scores are linked to a greater incidence of postoperative complications.^4^

Advanced age is linked to reduced physiological reserve, especially in the cardiopulmonary and renal systems, increasing the risk of perioperative respiratory complications. Older patients may have impaired responses to hypercapnia, decreased chest wall compliance, and lower pulmonary function. Age-related vascular stiffness and autonomic dysregulation can also worsen hemodynamic instability during procedures like steep Trendelenburg positioning or CO₂ insufflation.^5^ A longer hospital stay may indicate preexisting comorbidities, postoperative complications, or delayed recovery, which can elevate anaesthetic risks, including the potential for hospital-acquired infections. PACU admission often signals a need for closer monitoring due to intraoperative instability or high anaesthetic load, particularly in patients who underwent complex procedures or exhibited risk factors like hypothermia or hemodynamic lability.^2^

The increasing prevalence of RALP highlights the necessity for a comprehensive understanding of the anaesthetic challenges associated with this procedure. Identifying risk factors for anaesthetic complications is crucial. Focusing on modifiable perioperative risk factors can optimize anaesthetic care and improve postoperative outcomes.

Despite the physiological challenges of RALP, including steep Trendelenburg positioning and CO₂ pneumoperitoneum, the incidence of anaesthetic complications remains low in a high-volume center. We hypothesize that RALP can be performed safely with individualized perioperative care, as evidenced by the low rate of PACU admissions and anaesthetic complications.

This study aims to assess the safety of RALP by evaluating the incidence of anaesthetic complications and identifying independent risk factors. Additionally, the rates and duration of PACU admission were analyzed to support the assessment of perioperative anaesthetic safety in a high-volume surgical center.

## Methods

### Study Design and Participants

This study was designed as a retrospective observational analysis conducted at Ankara Bilkent City Hospital from 2019 to 2024. The study group consisted of 1020 patients who underwent RALP during this period. Patients who underwent combined procedures or had incomplete medical records were excluded from the analysis. Ankara Bilkent City Hospital, Medical Research Scientific and Ethical Evaluation Board No. 1 (TABED) approved the study (protocol number: TABED1-24-371, date: 03.07.2024).

### Data Collection

Data were retrieved from electronic medical records. The preoperative variables included age, gender, body mass index (BMI), ASA physical status, comorbidities (eg, hypertension, diabetes mellitus, CAD, chronic kidney disease, cerebrovascular accident, and COPD), smoking history, and abnormal laboratory findings. Intraoperative complications were also recorded. Additionally, postoperative outcomes were documented, including the occurrence of pulmonary, cardiac, or neurological complications, length of hospital stay, rate of admissions to the PACU, and duration of PACU stay.

### Statistical Analysis

Descriptive statistics for continuous variables were presented as mean ± standard deviation (SD), median, and interquartile range (IQR, 25^th^-75^th^ percentiles), while categorical variables were expressed as percentages. The Shapiro-Wilk test was used to assess the normality of data distribution. The Mann-Whitney U test compared continuous variables between two groups. Group comparisons were conducted using the chi-squared or Fisher’s exact tests, as appropriate for categorical variables. Independent risk factors associated with the development of anaesthetic complications were analyzed using multivariate logistic regression analysis. All statistical analyses were performed using IBM SPSS version 20 (Chicago, IL, USA), and a *P* value of <0.05 was considered statistically significant.

## Results

The study included 1020 patients who underwent RALP. The mean age of the patients was 65.01±6.34 years, with a minimum age of 38 and a maximum age of 83. The mean hospital stay duration was 6.92±3.28 days. Postoperatively, 47.9% (n = 489) of the patients were admitted to the PACU, with an average PACU stay of 9.95±3.95 hours.

Most patients had an ASA score of 2 (65.3%), while 13.8% were ASA I, 20.7% were ASA III, and 0.2% were ASA IV. The prevalence of comorbidities was as follows: diabetes mellitus in 24.2% of patients, hypertension in 41%, CAD in 18.4%, and COPD in 9.9%. Additionally, 61.6% of the patients had at least one comorbidity (Table 1).

Anaesthetic complications occurred in 4.4% (n = 45) of patients. A comparison between patients with and without anaesthetic complications revealed statistically significant differences in the variables. Patients with anaesthetic complications were significantly older (67.93±6.22 vs. 64.88±6.32 years, *P*=0.004). Patients with anaesthetic complications had significantly longer hospital stays (8.98±4.45 days vs. 6.83±3.18 days, *P* < 0.001). Patients who experienced anaesthetic complications had a higher rate of being monitored in the PACU (73.3% vs. 46.8%, *P* < 0.001). No significant differences were observed in PACU stay duration (4.64±4.94 vs. 3.91±3.88 hours, *P*=0.388), ASA scores (*P*=0.379), and comorbidity rates (68.9% vs. 61.2%, *P*=0.302) between patients with and without anaesthetic complications (Table 2).

Multivariate logistic regression analysis identified age, length of hospital stay, and PACU admission as independent risk factors for developing anaesthetic complications. Each 1-year increase in age increased the risk of anaesthetic complications by 1.083 times [odds ratio (OR): 1.083, 95% confidence interval (CI): 1.024-1.142, *P*=0.003]. Each additional day of hospital stay increased the risk by 1.128 times (OR: 1.128, 95% CI: 1.059-1.201, *P < 0.001*). Admission to the PACU was associated with a 3.363-fold increase in the risk of anaesthetic complications (OR: 3.362, 95% CI: 1.694-6.671, p=0.001; Table 3).

## Discussion

In our study of 1020 RALP patients, the mean age was 65 years with an average hospital stay of 6.9 days, with nearly half (47.9%) requiring postoperative PACU monitoring. Most patients were classified as ASA II and over 60% had at least one comorbidity. Anaesthetic complications occurred in 4.4% of cases; notably, patients with these complications were significantly older (mean age 67.93 vs. 64.88 years), had longer hospital stays (8.98 vs. 6.83 days), and were more frequently admitted to the PACU (73.3% vs.. 46.8%) compared with those without complications. Multivariate analysis identified age, length of hospital stay, and PACU admission as independent risk factors, with each additional year of age and day in the hospital increasing the risk by 1.083 and 1.128 times, respectively, while PACU admission tripled the risk of anaesthetic complications.

A retrospective study by Uğur and Ertuğrul^6^ analyzed anaesthesia management in robotic-assisted perineal prostatectomy (RAPP) cases and focused on physiological challenges related to steep trendelenburg positioning and pneumoperitoneum. The study included 131 patients and evaluated intraoperative hemodynamic and respiratory changes and postoperative complications. The findings indicate significant intraoperative decreases in heart rate, systolic and diastolic blood pressure, and mean arterial pressure. Additionally, CO_2_ pneumoperitoneum led to increased pCO_2_ levels and decreased pH, although no severe acid-base imbalances were observed. The most common postoperative complications were nausea and vomiting, followed by anastomotic leakage. The study highlights the importance of understanding the physiological effects of RAPP on older patients with comorbidities and emphasizes the need for careful anaesthesia management. The results contribute to optimizing perioperative strategies to mitigate complications associated with robot-assisted surgery.^6^

Porcaro et al.^7^ assessed the predictive value of the ASA physical status classification for 90-day postoperative complications after RALP. In their large cohort, higher ASA scores were independently associated with an increased risk of significant complications (as graded by the Clavien-Dindo system). This finding supports the use of ASA classification for preoperative risk stratification and underscores its utility in surgical planning and patient counseling.^7^

The study by Zhang et al.^8^ investigated the risk factors associated with hypoxemia during the emergence from anaesthesia in patients undergoing RALP. The study included 316 patients divided into hypoxemia (n = 134) and non-hypoxemia (n = 182) groups based on postoperative oxygen levels. The findings revealed that 38.9% of patients had low preoperative partial pressure O_2_. Several clinical parameters, including BMI, preoperative PaO_2_ levels, and a history of emphysema and pulmonary alveolar disease, were significantly associated with hypoxemia. The study concluded that these factors are crucial predictors of hypoxemia during the emergence from anaesthesia in patients with RALP. The authors emphasized the importance of preoperative assessment and managing these risk factors to mitigate postoperative hypoxemia and improve patient outcomes.^8^

Aceto et al.^5^ provided a narrative review focusing on the challenges of administering anaesthesia during robot-assisted surgery in older people. The authors emphasized that extreme patient positioning (eg, steep or reverse Trendelenburg) combined with pneumoperitoneum can significantly affect cardiovascular stability, lung mechanics, intracranial pressure, and ocular perfusion. The review advocates for tailored anaesthetic techniques and vigilant intraoperative monitoring to mitigate these risks, particularly in older patients with compromised cardiopulmonary reserve.^5^

Høyer et al.^9^examined lung-protective ventilation’s hemodynamic, renal, and hormonal effects (eg, low tidal volume with high PEEP) during RALP. Their randomized controlled trial in 24 patients revealed that while lung-protective strategies might transiently reduce renal function (evidenced by decreased creatinine clearance) and increase levels of vasoactive hormones, these effects are reversible. The study underscores the need to balance lung protection with these patients’ potential hemodynamic and renal consequences.^9^

Emir et al.^10^ compared RAPP with the conventional RALP approach. Their retrospective study found that although RAPP was associated with a more pronounced increase in blood CO_2_ levels due to perineal insufflation, respiratory mechanics were less adversely affected than in RALP. The results suggest that differences in surgical approach necessitate distinct anaesthetic considerations, particularly regarding CO_2_ management.^10^

Lestar et al.^11^ focused on the hemodynamic changes during RALP performed in an extreme (45°) Trendelenburg position. Their investigation in 16 patients with an ASA score of 1-2 showed a marked increase (two-fold to three-fold) in filling pressures (eg, central venous pressure and pulmonary capillary wedge pressure) without compromising cardiac output or gas exchange. However, lung compliance was significantly reduced. These findings highlight the circulatory adaptations required during steep Trendelenburg and the importance of close hemodynamic monitoring.^11^

Danic et al.^12^ reviewed anaesthesia considerations in a series of 1,500 cases of RALP. They reported low rates of anaesthesia-related complications overall, with corneal abrasions and airway management challenges being the most common issues. Their extensive experience emphasizes that while robot-assisted prostatectomy is generally safe from an anaesthetic standpoint, careful attention to patient positioning and airway management is crucial.^12^

Our study highlights the incidence of pulmonary complications in patients undergoing RALP and identifies several independent risk factors. The steep trendelenburg position and CO_2_ pneumoperitoneum adversely affect respiratory function, leading to complications such as atelectasis and the need for postoperative oxygen support. Our findings are consistent with previous studies that have reported similar complications following RALP.

Compared to other robotic-assisted procedures such as gynecologic or colorectal surgeries, RALP poses unique anaesthetic challenges due to the steep Trendelenburg position maintained for prolonged durations and the proximity of the surgical field to the diaphragm and airway structures. For example, robotic hysterectomy and colorectal resections often require less extreme positioning and shorter pneumoperitoneum duration, potentially resulting in fewer cardiopulmonary perturbations. Moreover, RALP is typically performed in elderly male patients with multiple comorbidities, which further increases anaesthetic risk. These factors highlight that anaesthetic management in RALP must be especially vigilant regarding ventilation strategies, hemodynamic monitoring, and airway protection. Our findings reinforce these concerns, as older age and PACU admission were identified as independent predictors of anaesthetic complications in this specific surgical context.^13, 14, 15^

While our study focused on short-term perioperative outcomes, including hospital stay, PACU admission, and immediate anaesthetic complications, we acknowledge the importance of long-term consequences such as health-related quality of life, postoperative functional status, and long-term morbidity. Prior studies have shown that anaesthetic complications—especially pulmonary and cardiovascular events—can negatively impact rehabilitation, delay return to baseline functional capacity, and increase readmission or long-term dependency on healthcare.^16, 17, 18^ Future prospective studies with structured follow-up protocols should evaluate the long-term effects of anaesthetic events, including impacts on neurocognitive function, frailty progression, and quality of life in elderly patients undergoing RALP.

### Study Limitations

This study has several limitations that must be acknowledged. First, its retrospective design inherently limits the control over confounding variables and depends on the accuracy and completeness of existing medical records. Second, as the study was conducted at a single tertiary center with experienced surgical and anaesthetic teams, the findings may not be generalizable to institutions with different levels of expertise or perioperative protocols. Another significant limitation of our study is the lack of detailed intraoperative data, such as surgical duration, specific anaesthetic techniques, induction and maintenance agents, ventilation strategies, and surgical complexity scores. These variables are known to influence anaesthetic risk and perioperative outcomes. However, as a retrospective analysis relying on electronic medical records, such granular data were not consistently documented and thus could not be included in the statistical models. Variations in anaesthetic techniques, postoperative care models, and institutional practices can influence complication rates and outcomes. Future prospective studies should incorporate standardized intraoperative documentation, including real-time recording of anaesthetic protocols and procedural complexity, to better delineate their role in developing anaesthetic complications.

## Conclusion

In this large retrospective cohort of 1,020 patients undergoing RALP, we identified age, prolonged hospital stay, and PACU admission as independent predictors of anaesthetic complications. Despite the physiological demands imposed by steep Trendelenburg positioning and CO₂ pneumoperitoneum, the overall incidence of anaesthetic complications remained low. These findings support our initial hypothesis that RALP can be safely performed in high-volume centers with experienced perioperative teams and individualized anaesthetic planning.

This study adds to the growing evidence on perioperative safety in robotic urologic surgery by providing real-world data on risk stratification and complication rates. Importantly, it underscores the clinical relevance of proactively identifying vulnerable patients and implementing targeted anaesthetic strategies such as optimized ventilation, hemodynamic monitoring, and early PACU triage to reduce adverse events.

Future prospective multicenter trials with standardized intraoperative data collection are warranted to confirm these findings and to evaluate the long-term consequences of anaesthetic complications, including their impact on postoperative recovery, functional capacity, and quality of life.

## Ethics

**Ethics Committee Approval:** Ankara Bilkent City Hospital, Medical Research Scientific and Ethical Evaluation Board No. 1 (TABED) approved the study (protocol number: TABED1-24-371, date: 03.07.2024).

**Informed Consent:** All participants provided informed consent.

## Figures and Tables

**Table 1. Patient Characteristics:** 

**n = 1020**	**Mean ± SD; median (min.-max.); IQR**	
**Age (years)**	65.01±6.34; 65 (38-83); (61-69)	
**Hospital stay (days)**	6.92±3.28; 6 (3-32); (5-8)	
**PACU stay duration (hours) n = 489**	9.95±3.95; 2.5 (0.5-24.0); (1.5-4.5)	
-	**n**	**%**
**ASA score**		
1	140	13.8
2	665	65.3
3	211	20.7
4	2	0.2
**PACU admission**		
No	531	52.1
Yes	489	47.9
**Diabetes mellitus**		
No	773	75.8
Yes	247	24.2
**Hypertension**		
No	602	59.0
Yes	418	41.0
**Coronary artery disease**		
No	832	81.6
Yes	188	18.4
**Chronic obstructive pulmonary disease**		
No	919	90.1
Yes	101	9.9
Comorbidities*		
No	392	38.4
Yes	628	61.6
**Anaesthetic complications**		
No	975	95.6
Yes	45	4.4

*Including diabetes mellitus, hypertension, coronary artery disease, and chronic obstructive pulmonary disease.

ASA, American Society of Anesthesiologists; IQR, interquartile range; PACU, post-anaesthesia care unit; SD, standard deviation; min.-max., minimum-maximum

**Table 2. Comparison of Patients with and without Anaesthetic Complications:** 

-	**No anaesthetic complications (n = 975)**		**Anaesthetic complications (n = 45)**		***P* value**
	Mean ± SD/median (IQR)		Mean ± SD/median (IQR)		-
**Age (years)**	64.88±6.32 65 (61-69)		67.93±6.22 68 (63-73)		**0.004^b^**
**Hospital stay (days)**	6.83±3.18 6 (5-8)		8.98±4.45 8 (6-11)		**<0.001^b^**
**PACU stay duration (hours; n = 489)**	3.91±3.88 2.5 (1.5-4.5)		4.64±4.94 3 (1.7-5.5)		0.388^b^
-	**n**	**%**	**n**	**%**	-
**ASA score**					
1	136	14.0	4	8.9	0.379^c^
2	637	65.5	28	62.2	
3	198	20.3	13	28.9	
4	2	0.2	0	0	
**PACU admission**					
No	519	53.2	12	26.7	**<0.001^c^**
Yes	456	46.8	33	73.3	
**Comorbidities**					
No	378	38.8	14	31.1	0.302^c^
Yes	579	61.2	31	68.9	

^b^, Mann-Whitney U test; ^c^, chi-squared test/Fisher’s exact test.

ASA, American Society of Anesthesiologists; IQR, interquartile range; PACU, post-anaesthesia care unit; SD, standard deviation.

**Table 3. Multivariate Logistic Regression Analysis of Risk Factors for Anaesthetic Complications:** 

**Variable**	**B (SE)**	**Adjusted OR**	**95% CI**	***P* value**
Age	0.080 (0.027)	1.083	1.024-1.142	0.003
Hospital stay (days)	0.120 (0.032)	1.128	1.059-1.201	<0.001
PACU admission	1.213 (0.350)	3.362	1.694-6.671	0.001

CI, confidence interval; OR, odds ratio; PACU, post-anaesthesia care unit; SE, standard error.

## References

[1] Novara G, Ficarra V, Rosen RC (2012). Systematic review and meta-analysis of perioperative outcomes and complications after robot-assisted radical prostatectomy.. Eur Urol.

[2] De Carlo F, Celestino F, Verri C, Masedu F, Liberati E, Di Stasi SM (2014). Retropubic, laparoscopic, and robot-assisted radical prostatectomy: surgical, oncological, and functional outcomes: a systematic review.. Urol Int.

[3] Gainsburg DM (2012). Anesthetic concerns for robotic-assisted laparoscopic radical prostatectomy.. Minerva Anestesiologica.

[4] Awad H, Walker CM, Shaikh M, Dimitrova GT, Abaza R, O'Hara J (2012). Anesthetic considerations for robotic prostatectomy: a review of the literature.. J Clin Anesth.

[5] Aceto P, Galletta C, Cambise C (2023). Challenges for anaesthesia for robotic-assisted surgery in the elderly: a narrative review.. Eur J Anaesthesiol Intensive Care.

[6] Uğur Ö, Ertuğrul F (2023). Retrospective analysing of anesthesia manegement in robotic assisted radical prostatectomy cases.. J Cukurova Anesth Surg.

[7] Porcaro AB, Rizzetto R, Bianchi A (2023). American Society of Anesthesiologists (ASA) physical status system predicts the risk of postoperative Clavien–Dindo complications greater than one at 90 days after robot-assisted radical prostatectomy: final results of a tertiary referral center.. J Robot Surg.

[8] Zhang Q, Zhu L, Yuan S, Lu S, Zhang X (2024). Identifying risk factors for hypoxemia during emergence from anesthesia in patients undergoing robot-assisted laparoscopic radical prostatectomy.. J Robot Surg.

[9] Høyer S, Mose FH, Ekeløf P, Jensen JB, Bech JN (2021). Hemodynamic, renal and hormonal effects of lung protective ventilation during robot-assisted radical prostatectomy, analysis of secondary outcomes from a randomized controlled trial.. BMC Anesthesiol.

[10] Emir NS, Akyol D, Sabaz MS, Karadağ S (2023). Robotic assited perineal prostatectomy (RAPP) as a new era for anesthesiology: It’s effects on hemodynamic parameters and respiratory mechanics.. J Robot Surg.

[11] Lestar M, Gunnarsson L, Lagerstrand L, Wiklund P, Odeberg-Wernerman S (2011). Hemodynamic perturbations during robot-assisted laparoscopic radical prostatectomy in 45 trendelenburg position.. Anesth Analg.

[12] Danic MJ, Chow M, Alexander G, Bhandari A, Menon M, Brown M (2007). Anesthesia considerations for robotic-assisted laparoscopic prostatectomy: a review of 1,500 cases.. J Robot Surg.

[13] Watrowski R, Kostov S, Alkatout I (2021). Complications in laparoscopic and robotic-assisted surgery: definitions, classifications, incidence and risk factors - an up-to-date review.. Wideochir Inne Tech Maloinwazyjne.

[14] Parsad S, Sharma A, Jangra K, Kumar S, Sharma G (2021). Anaesthesia concerns of steep trendelenburg position in robotic pelvic surgeries: a critical review.. IJCA.

[15] Popescu M, Olita MR, Stefan MO, Mihaila M, Sima RM, Tomescu D (2022). Lung mechanics during video-assisted abdominal surgery in trendelenburg position: a cross-sectional propensity-matched comparison between classic laparoscopy and robotic-assisted surgery.. BMC Anesthesiol.

[16] Brzezinski M, Rooke GA, Azocar RJ. Anesthetic management. In: Rosenthal R, Zenilman M, Katlic M, eds. Principles and Practice of Geriatric Surgery. Springer, Cham; 2020

[17] Lin HS, McBride RL, Hubbard RE (2018). Frailty and anesthesia – risks during and post-surgery.. Local Reg Anesth.

[18] Damluji AA, Forman DE, van Diepen S (2020). ; American Heart Association Council on Clinical Cardiology and Council on Cardiovascular and Stroke Nursing. Older adults in the cardiac intensive care unit: factoring geriatric syndromes in the management, prognosis, and process of care: a scientific statement from the American Heart Association.. Circulation.

